# Oral and topical peptides for skin aging: systematic review and meta-analysis of randomized controlled trials

**DOI:** 10.3389/fmed.2026.1618306

**Published:** 2026-03-17

**Authors:** Houriah Y. Nukaly, Ibrahim R. Halawani, H. M. Irtaza, Talah Alturkistani, Mohammed Rehab Serafi, Waseem Alhawsawi, Hassan Omar Bogari, Ferdous A. Ahmed, Yara Alhaddad, Asem Shadid, Ruaa Alharithy, Abdulhadi Jfri

**Affiliations:** 1 Division of Experimental Medicine, McGill University Health Centre, Montréal, QC, Canada; 2 Faculty of Medicine, King Abdulaziz University, Jeddah, Saudi Arabia; 3 Department of Medicine, Bahawal Victoria Hospital, Bahawalpur, Pakistan; 4 King Saud Bin Abdulaziz University for Health Sciences, Jeddah, Saudi Arabia; 5 Department of Dermatology, King Fahad Armed Forces Hospital, Jeddah, Saudi Arabia; 6 Batterjee Medical College for Sciences and Technology, Jeddah, Saudi Arabia; 7 Department of Dermatology, King Fahad Medical City, Riyadh, Saudi Arabia; 8 Security Forces Hospital, Riyadh, Saudi Arabia; 9 Princess Nourah University, Riyadh, Saudi Arabia; 10 College of Medicine, King Saud bin Abdulaziz University for Health Sciences, Jeddah, Saudi Arabia; 11 King Abdullah International Medical Research Center, Jeddah, Saudi Arabia

**Keywords:** skin aging interventions treatments, collagen peptides, oral peptides, skin aging, topical peptides

## Abstract

**Introduction:**

Skin aging manifests as wrinkles, reduced elasticity, and roughness due to intrinsic and extrinsic factors. Peptide-based therapies enhance collagen synthesis and extracellular matrix integrity. This systematic review and meta-analysis (SRMA) evaluates the efficacy and safety of oral and topical peptides in improving hydration, elasticity, wrinkles, and brightness.

**Methods:**

A comprehensive search of MEDLINE, CENTRAL, and Web of Science was conducted following PRISMA guidelines. Randomized controlled trials (RCTs) assessing peptide effects on skin aging parameters were included. The Cochrane Risk of Bias Tool (RoB 2) assessed study quality. Data were synthesized using a random-effects model in RStudio (R version 4.1.1).

**Conclusion:**

Nineteen RCTs involving 1,341 participants were analyzed. Peptides, particularly oral formulations, significantly improved hydration and brightness, with a modest pooled effect on wrinkle reduction (MD = 0.27, *p* = 0.04). Subgroup analysis indicated that this benefit was largely driven by oral polypeptides (MD = 1.5, *p* = 0.01). While effects on elasticity and density were inconsistent, peptides were well tolerated, with minimal adverse events reported across trials. Overall, peptides appear to be safe, non-invasive anti-aging agents, though larger RCTs with standardized outcomes and histopathologic assessment are warranted.

**Systematic review registration:**

identifier CRD420250652779.

## Highlights

- Oral polypeptides significantly improve skin hydration (Mean Difference [MD]: 5.80, *p* < 0.01) and wrinkle reduction (MD: 0.35, *p* = 0.05), outperforming topical formulations.
- Peptides enhance skin brightness (MD: 2.40, *p* = < 0.01) but show minimal effects on elasticity.

- Peptides are well-tolerated with minimal adverse events, supporting their role as safe, non-invasive anti-aging agents.

## Introduction

The phenomenon of aging is inescapable and is characterized by multiple clinical manifestations, including wrinkles, reduced skin density, loss of elasticity, xerosis, uneven pigmentation, telangiectasia, sagging, and impaired wound healing. The intrinsic aging process is compounded by extrinsic factors such as UV exposure, environmental pollution, and lifestyle choices (e.g., smoking, alcohol abuse, unhealthy diet), thus, leading to accelerated degradation of collagen and elastin fibers. The disruption of these proteins contributes to the visible signs of aging, significantly impacting individuals' aesthetic appearance and psychological wellbeing. Certain dermatological conditions, such as actinic elastosis and mid-dermal elastolysis, share overlapping clinical features with photoaging, particularly wrinkling, although they arise through distinct pathological mechanisms (1).

Recent advancements have introduced orally administered or topically applied biologically active compounds, such as collagen peptide-based supplements (2–4), which have shown promising effects in supporting skin rejuvenation and represent a safe, non-invasive adjunct to conventional anti-aging strategies. These oligopeptides are obtained by enzymatic hydrolysis of natural collagen. The mechanism by which peptides enhance cutaneous collagen is multifaceted. Peptides act as signaling molecules that mimic the body's natural processes to stimulate collagen synthesis, as well as enhancing other extracellular matrix (ECM) components such as hyaluronic acid by fibroblasts, help to rebuild the skin's structural integrity, resulting in improved skin texture, reduced wrinkles, and increased elasticity and hydration.

Peptides can be broadly categorized into several functional classes based on their mechanisms of action. Signal peptides stimulate fibroblast activity and collagen synthesis, thereby improving dermal structure and resilience. Carrier peptides deliver trace elements such as copper, which are essential cofactors in enzymatic processes involved in wound healing and skin repair. Neurotransmitter-inhibitory peptides act by modulating neuromuscular activity to reduce dynamic wrinkles. In contrast, enzyme-inhibitory peptides protect the extracellular matrix by blocking enzymes such as collagenase and elastase that degrade structural proteins. This mechanistic diversity underscores the rationale for evaluating peptide-based therapies in skin rejuvenation, as each class contributes distinct yet complementary effects on the aging skin (2–4). However, their clinical effectiveness is shaped by important pharmacokinetic considerations: topical formulations face the barrier of stratum corneum penetration, which is highly dependent on peptide size, hydrophobicity, and vehicle composition, while oral peptides are subject to gastrointestinal degradation and variable absorption rates. These limitations highlight the need for optimized formulations and delivery strategies to achieve consistent and clinically meaningful outcomes (3, 4).

A prime example of the efficacy of oral supplements is hydrolyzed collagen peptides, that significantly improved skin hydration, elasticity, roughness, and density in a randomized, placebo-controlled trial (2). Topical applications of peptide formulations also offer a targeted approach to skin rejuvenation. For instance, a cream containing Matrixyl (a peptide compound) demonstrated a significant reduction in wrinkle volume and depth (4).

Despite a growing body of evidence supporting the efficacy of collagen peptides in reducing age-related skin changes, a consolidated review analyzing their impact across various studies is necessary to validate these findings and guide future clinical practices. This systematic review and meta-analysis of randomized controlled trials (RCTs) focuses on adult patients of varying ages, genders, and skin types presenting with signs of aging, such as wrinkles, reduced elasticity, and skin roughness, who received peptide-based therapies for anti-aging effects. The primary objective was to evaluate the efficacy of different types of peptides in improving skin hydration, elasticity, and wrinkle reduction, assess the adverse events associated with peptide-based treatments, and determine the effectiveness of oral VS. topical peptide formulations for various skin aging parameters.

## Materials and methods

### Literature search and study selection

A systematic literature search was conducted using predefined search terms: (“peptides” OR “anti-aging” OR “skin rejuvenation” OR “wrinkle reduction”) AND (“Randomized Controlled Trials” OR “RCTs”). Databases searched included MEDLINE, Web of Science, and CENTRAL (Table 1). The study protocol was registered in PROSPERO (Registration ID: CRD420250652779) and adhered to the PRISMA guidelines (5). Initial screening of articles based on titles and abstracts was followed by the removal of duplicates. Full-text reviews were independently conducted for potentially relevant studies, adhering strictly to predetermined inclusion and exclusion criteria.

**Table 1:** Database search strategy.

**Database**	**Search terms**	**Records identified (*n*)**	**Filters applied**	**Search period**
MEDLINE (PubMed and OVID)	(“peptides” OR “collagen peptides” OR “signal peptides” OR “oligopeptides” OR “tripeptides”) AND (“anti-aging” OR “skin rejuvenation” OR “wrinkle reduction” OR “skin hydration” OR “skin elasticity” OR “skin brightness”) AND (“randomized controlled trial” OR “RCT”)	1,680	Humans only; English language	From inception to February 2025
CENTRAL (Cochrane Library)	(“peptides” OR “collagen peptides” OR “signal peptides” OR “topical peptides” OR “oral peptides”) AND (“skin aging” OR “wrinkle reduction” OR “skin elasticity” OR “hydration” OR “brightness”)	272	Humans only; English language	From inception to February 2025
Web of Science (WOS)	(“peptide”^*^ AND “anti-aging” AND “skin”) AND (“randomized controlled trial” OR “clinical trial”)	100	Humans only; English language	From inception to February 2025

### Inclusion and exclusion criteria

Eligible studies included RCTs involving adult participants with signs of skin aging. Studies evaluating the anti-aging effects of peptides delivered via oral or topical routes and reporting quantitative outcomes such as wrinkle reduction, hydration, brightness, elasticity, and skin roughness were included. Studies with incomplete descriptions of treatment protocols, non-English publications, observational studies, narrative reviews, meta-analyses, systematic reviews, and studies involving concurrent anti-aging interventions were excluded. Additional exclusions applied to animal, *in-vitro*, or cadaveric studies, as well as case series, case reports, or editorials (Table 2).

**Table 2:** PICO framework.

**Component**	**Description**
Population (P)	Adult participants with clinical signs of skin aging, including wrinkles, loss of elasticity, dryness, and dullness. • Mean age across trials ranged approximately from the early 40s to early 80s (pooled mean age, calculated: 50.2 ± 9.1 years). • Total sample size: 1,341 participants across 19 randomized controlled trials (2001–2024). • Geographic distribution: Asia (Japan, Korea, China, Thailand), Europe (Germany, Italy), North America (USA), South America (Brazil). • Fitzpatrick skin types II-V were represented, though underreported in several trials.
Intervention/Exposure (I)	Administration of peptide-based anti-aging therapies, either: • Oral formulations: collagen tripeptides, hydrolyzed collagen peptides, oligopeptides, or composite nutraceuticals containing amino acids, vitamins, or minerals. • Topical formulations: signal peptides (e.g., Argireline, Matrixyl), enzyme-inhibitory or defensin-based creams or serums. Intervention duration: 4-12 weeks.
Comparison (C)	Placebo or vehicle-control treatments (e.g., maltodextrin, cellulose capsules, or identical base creams). Comparisons also included baseline vs. post-intervention assessments of skin hydration, elasticity, roughness, wrinkles, and brightness.
Outcomes (O)	Primary Outcomes: • Improvement in wrinkle reduction (Mean Difference [MD] = 0.27; *p* = 0.04; oral subgroup MD = 1.5; *p* = 0.01). • Skin hydration increase (MD = 5.80; *p* < 0.01). Secondary Outcomes: • Skin brightness improvement (MD = 2.40; *p* ≤ 0.01). • Reduction in skin roughness (MD = −8.47; *p* = 0.05). • Minimal change in elasticity (MD = 0.09; *p* = 0.15). • Safety outcomes: no severe adverse events; mild gastrointestinal discomfort reported in isolated cases.
Study design	Systematic review and meta-analysis of 19 randomized, double-blind, placebo-controlled trials, conducted in accordance with PRISMA 2020 guidelines and registered in PROSPERO (CRD420250652779). Risk of bias assessed using the Cochrane RoB 2 tool.
Effect estimates (key results)	•Peptides significantly improved hydration and wrinkle reduction, with effects predominantly driven by oral formulations. • Brightness improved significantly; elasticity showed modest, non-significant changes. • High heterogeneity (*I*^2^ ≈ 100%) attributed to variability in peptide type, dose, and assessment methods. • Subgroup analysis: oral peptides outperformed topical formulations across most outcomes.
Conclusion	Oral and topical peptides provide modest but significant improvements in key skin-aging parameters, especially hydration and brightness, with a strong safety profile. However, limited standardization of formulations and imbalance between oral (*n* = 17) and topical (*n* = 2) studies constrain definitive conclusions.
Clinical implication	Peptides represent a safe, non-invasive adjunct for skin rejuvenation and anti-aging therapy. Oral collagen-based peptides may offer systemic benefits, while optimized topical formulations could provide localized effects. Future studies should include standardized endpoints, histopathologic validation, and balanced comparisons of administration routes.

### Screening and data extraction

Screening and review were conducted by two teams of reviewers: Group one (HB and TT) and Group two (IH and MS), using Rayyan software (6) for title and abstract screening. Each author independently screened titles, abstracts, and full texts for inclusion. Discrepancies in study selection were resolved by consensus with senior authors (AJ, RA). Full-text screening followed the same process. Data extraction was performed using Excel spreadsheets, capturing essential study details such as sample size, mean age, gender, race, intervention type (oral or topical peptides), peptide source and concentration, frequency and duration of administration, primary outcomes (hydration, elasticity, wrinkle reduction), and adverse events. Information on peptide molecular weight, classification, and whether peptides were used alone or in combination with other active compounds was not consistently reported in the included trials and, therefore, could not be systematically extracted.

### Assessment of methodological quality

Two independent reviewers assessed the methodological quality of included RCTs using the Cochrane Risk of Bias Tool 2 (RoB 2) (7). It categorizes bias within each domain as low, high, or having some concerns without assigning a cumulative score. Key domains assessed included randomization, allocation concealment, blinding, and outcome reporting. Discrepancies between reviewers were resolved by senior authors (AJ, RA).

### Data synthesis and analysis

The entire review process is illustrated in a PRISMA flowchart. Data were synthesized using Rstudio (R version 4.1.1). A random-effects model was applied to account for variability across studies, with forest plots used to visualize pooled results. Heterogeneity was assessed using the *I*^2^ statistic, with values >50% considered indicative of substantial heterogeneity. Both fixed- and random-effects models were applied in sensitivity analyses. Subgroup analyses were performed to compare oral vs. topical peptides and to explore variations based on peptide type and concentration. In addition, a leave-one-out sensitivity analysis was conducted to evaluate the influence of individual studies on pooled estimates and heterogeneity. Further subgroup analyses based on molecular weight, peptide classification, or co-formulation with other active compounds were not feasible due to insufficient reporting in the primary studies.

## Results

### Literature findings

**Figure 1:**
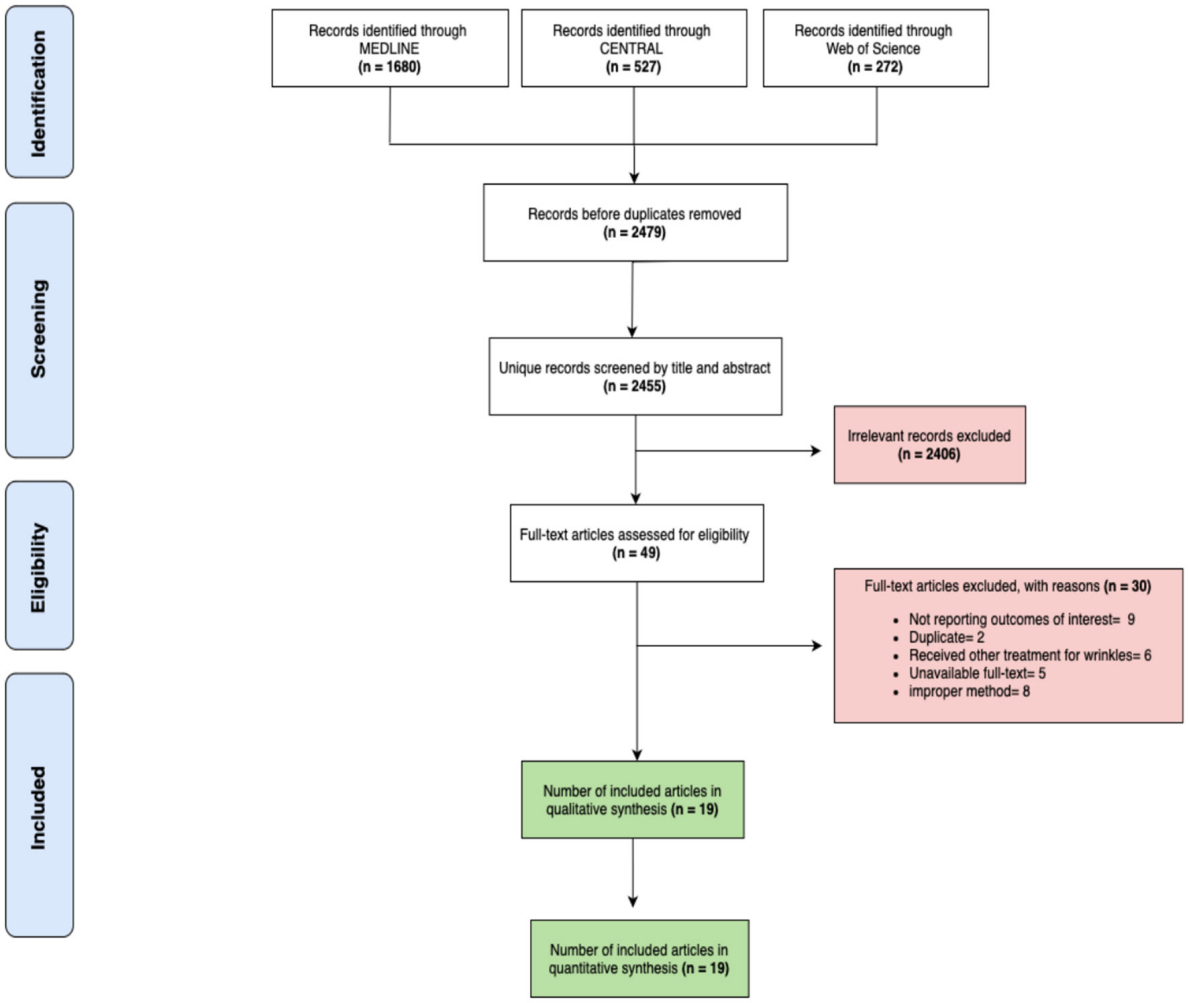
The flowchart of the reviewed studies according to PRISMA, representing the full process of article inclusion.

A systematic review of the literature identified a total of 2,479 articles retrieved from the initial search, distributed across various databases: 1,680 from MEDLINE, 527 from CENTRAL, and 272 from Web of Science (WOS). After the removal of duplicates, 2,455 articles remained for screening. A comprehensive review process led to the selection of 49 articles for full-text screening, of which 19 met the eligibility criteria and were included in the final analysis. Several articles were excluded for reasons such as language limitations, inappropriate methodology, or lack of available data. Ultimately, these 19 articles were included in the final analysis to assess the effectiveness of peptide-based therapies, as illustrated in the PRISMA flow diagram (Figure 1).

### Characteristics of included studies

The 19 included studies were RCTs evaluating the effectiveness of various peptide-based therapies across diverse populations. Of these, 17 studies investigated oral peptide formulations, while two studies evaluated topical formulations. The included participants had an age range from early 20s to mid-80s, covering both male and female demographics. Conducted in various countries, the interventions in these trials involved different peptide compounds administered through methods such as oral or topical application. The primary outcomes measured across these studies included parameters such as skin hydration, elasticity, wrinkle reduction, skin density, and biophysical skin properties. All trials employed a double-blind, placebo-controlled design, with treatment durations varying from 4 to 12 weeks, and follow-up consistently reported across studies as summarized in (Table 3).

**Table 3:** Characteristics of the included studies.

**No**.	**Study (author, year)**	**Intervention**	**Country**	**Sample**	**Mean age (years)**	**Gender (male/ female)**	**Wrinkle reduction**
1	Lin et al. (26)	Oral Supplementation	Taiwan	25	NA	0/50	Wrinkle: 88% improved (−29.3%) Crow's feet: 100% improved (−14.9%)
		Placebo		25			Wrinkle: 48% improved (+8.9%) Crow's feet: 80% improved (−7.7%)
2	Murad and Tabibian (27)	Oral anti-oxidant supplement	USA	53	46.1 ± 6.7	0/65	34% reduction in number of visible wrinkles (silfo replicas; *P* < 0.01) in treated group; no significant changes in controls
		Control		12			
3	Schwartz (28)	Hydrolyzed Collagen	USA	58	51.15 ± 5.32	0/113	Significant reduction in facial lines and wrinkles (*P* = 0.019) and crow's feet lines and wrinkles (*P* = 0.05)
		Control		55	50.87 ± 5.62		NR
4	Nomoto and Iizaka (29)	Once-a-day Administration of the ONS Containing Collagen Peptides	Japan	20	82.5 ± 6.4	6/14	NR
		Control		19	78.5 ± 7.1	6/13	NR
5	Petersen Vitello Kalil et al. (30)	Ortho-silicic acid stabilized by hydrolyzed marine collagen	Brazil	11	45.6 ± 4.8	1/10	NA (clinical evaluations showed significant improvement in texture, firmness, and hydration vs placebo; objective wrinkle counts on VISIA showed no significant between-group differences)
		Placebo		11	46.1 ± 3.7	2/9	NR
6	Proksch et al. (24)	Bioactive collagen peptide (Specific bioactive collagen peptides (derived from porcine skin collagen)	Brazil	57	55.6 ± 5.7	0/114	Significant reduction in eye wrinkle volume vs. placebo after 4 weeks and 8 weeks (~20%); maximum reduction 49.9%; effect persisted 4 weeks after stopping treatment (*P* < 0.05)
		Placebo (maltodextrin)		57	55.6 ± 6.2		NR
7	Proksch et al. (31)	Collagen hydrolysate 2.5	Germany	23	48.7 ± 4.8	0/69	NR
		Collagen Hydrolase 5.0		23	47.2 ± 5.7		NR
		Placebo		23	47.9 ± 5.2		NR
8	Sangsuwan and Asawanonda (32)	Collagen Hydrolysate	Thailand	17	55.8 ± 2.8	0/36	NR
		Placebo		19	56.4 ± 2.9		NR
9	Seong et al. (33)	Low-Molecular-Weight Collagen Peptides	South Korea	45	45.27 ± 6.36	12/33	NR
		Placebo		42	43.71 ± 6.0	12/30	NR
10	Tak et al. (11)	Oral Collagen Tripeptides	South Korea	41	48.0 ± 5.9	0/81	Significant improvement in skin wrinkling parameters (including visual grade of left/right crow's-feet) in CTP group vs placebo at 12 weeks; TEWL significantly reduced vs placebo (*P* < 0.05)
		Placebo		41	49.9 ± 6.5		NR
11	Wang et al. (34)	Topical Argireline (acetyl hexapeptide-3)	China	45	43.7 ± 7.5	7/38	48.90%
		Placebo		15	41.3 ± 8.3	3/12	0%
12	Bolke et al. (9)	Hydrolyzed collagen peptides	Germany	36	50.6 ± 11.2	0/72	Skin roughness significantly improved in intervention vs placebo (objective PRIMOS measurements) after 12 weeks (*P* < 0.05); improvements retained during 4-week follow-up.
		Placebo		36	52.4 ± 8.3		NR
13	Czajka et al. (8)	Oral hydrolyzed fish collagen (type I) with added micronutrients (vitamin C, zinc, copper, hyaluronic acid, etc.) liquid nutraceutical	Italy	61	43 ± 13.1	11/50	Fine lines improved in crow's feet and nasolabial folds clinically/photographically; DermaVision analysis showed wrinkle reductions in representative subjects (data not shown): crow's feet −4.5% and −3.8%; nasolabial folds −4.5% and −5.3%.
		Placebo		59	43 ± 12.3	18/41	NR
14	Inoue et al. (3)	Oral collagen hydrolysate with low content of bioactive dipeptides (L-CP)	China	28	43.25 ± 4.06	0/80	H-CP showed significantly greater improvement vs placebo in facial aging signs including wrinkles and roughness at 4 and 8 weeks; L-CP showed improvement but less than H-CP.
		High content (H-CP)		26	42.31 ± 4.92		
		Placebo (maltodextrin)		26	42.31 ± 4.80		
15	Ito et al. (35)	CPO	Japan	10	40.0 ± 6.8	4/17	NR
		Placebo		11	40.4 ± 5.2		
16	Kim et al. (10)	Low-molecular-weight Collagen Peptide	Korea	33	48.00 ± 4.50	0/64	After 12 weeks, crow's-feet visual grade significantly improved in LMWCP vs placebo (*p* = 0.013). Instrumental wrinkling parameters R1, R3, and R4 significantly improved vs placebo at week 12 (*p* = 0.043, 0.025, 0.004, respectively).
		Placebo		31	48.35 ± 4.32		NR
17	Miyeong et al. (36)	CPNS	Korea	54	45.7 +- 7.5	0/54	PRIMOS 3D wrinkling significantly improved vs placebo with reductions in Ra, Rmax, Rz, Rp, and Rv (μm) at 12 weeks Mean reduction from baseline to Week 12 (Δ, μm): Ra: −1.17 ± 0.32 (CPNS) vs. −0.44 ± 0.22 (Placebo) Rmax: −7.03 ± 2.14 (CPNS) vs. −1.71 ± 1.97 (Placebo) Rz: −5.44 ± 1.73 (CPNS) vs. −1.67 ± 1.73 (Placebo) Rp: −3.74 ± 1.23 (CPNS) vs. −1.04 ± 1.12 (Placebo) Rv: −3.78 ± 1.20 (CPNS) vs. −0.99 ± 1.34 (Placebo)
		Placebo		46	44.9 +- 5.2	0/46	
18	Taub et al. (37)	Placebo	USA	15	60	0/15	Significant reduction in visible pores, superficial wrinkles, and deep wrinkle; Wrinkle reduction: Using the Griffiths wrinkle grading scale (0–4; higher score = worse wrinkles), the analysis included only participants with moderate-to-severe superficial wrinkles at baseline (score 2–4).
		Alpha-defensin 5 + beta-defensin 3		31		0/31	At week 6 or week 12, 21/25 (84%) participants in the full formula group (α-defensin-5 + β-defensin-3 regimen) achieved ≥1-grade improvement in superficial wrinkles, compared with 6/12 (50%) in the placebo group (P = 0.048).
19	Kim et al. (38)	Collagen Placebo	Korea	50 50		3/40 5/36	NR
**No**	**Article**	**Route**	**Peptide type**		**Peptide formulation**	**Source**	**Dose (if oral)**
1	Lin et al. (26)	Oral	Hydrolyzed fish collagen combined with Djulis extract		Oral hydrolyzed fish collagen combined with Djulis extract	Fish collagen (pangasius, tilapia, cod, catfish, haddock) + Djulis (Chenopodium formosanum) extract	~5.5 g collagen + 1 g Djulis per day (50 mL drink)
			Placebo drink		–	50 mL of water and 3% apple juice	50 mL
2	Murad and Tabibian (27)	Oral	N-acetyl D-glucosamine and glucosamine sulfate		Oral supplement tablets (nutraceutical) containing N-acetyl-D-glucosamine, glucosamine sulfate, amino acids, minerals, antioxidants	Synthetic/ nutraceutical (non-collagen formulation)	4 tablets daily (dose not standardized in mg)
			Placebo group did not receive the supplement		–	–	–
3	Schwartz, 2019 [23]	Oral	Hydrolyzed collagen type II with glycosaminoglycans		Oral hydrolyzed collagen type II with chondroitin sulfate and hyaluronic acid	Chicken sternal cartilage	500 mg twice daily (300 mg collagen, 100 mg chondroitin sulfate, 50 mg hyaluronic acid)
			Placebo group received cellulose capsules.		–	–	–
4	Nomoto and Iizaka (29)	Oral	Collagen peptides		Oral collagen peptide supplement	Fish-derived collagen	10 g/day in 125 mL supplement
			Placebo: Standard hospital diet (no peptide supplement)		–	–	–
5	Petersen Vitello Kalil et al. (30)	Oral	Hydrolyzed collagen stabilized with ortho-silicic acid		Oral hydrolyzed marine collagen with ortho-silicic acid	Marine collagen	600 mg/day
			Placebo: Microcrystalline cellulose		–	–	–
6	Proksch et al. (24)	Oral	Specific Bioactive Collagen Peptides (BCP)		Oral bioactive collagen peptides	Porcine skin collagen	2.5 g/day
			Placebo: Maltodextrin		–	–	–
7	Proksch et al. (31)	Oral	Collagen hydrolysate (CH) composed of specific collagen peptides		Specific bioactive collagen peptides (derived from porcine skin collagen)	Porcine skin collagen	2.5 g/day
			Collagen hydrolysate (CH) composed of specific collagen peptides		Specific bioactive collagen peptides (derived from porcine skin collagen)	Porcine skin collagen	5 g/day
			Placebo: Maltodextrin		–	–	–
8	Sangsuwan and Asawanonda (32)	Oral	Collagen hydrolysate (CH)		Oral fish-derived collagen hydrolysate	Fish scale and skin collagen	5 g/day
			Placebo: Maltodextrin		–	–	–
9	Seong et al. (33)	Oral	Low-Molecular-Weight Collagen Peptides		Oral low-molecular-weight collagen peptides (fish-derived)	Nile tilapia fish scales	2.5 g/day
			Placebo: Maltodextrin-based supplement without active peptides		–	–	–
10	Tak et al. (11)	Oral	Collagen Tripeptide (CTP)		Collagen Tripeptide	Nile tilapia skin	1 g/day
			Placebo: Maltodextrin and Dextrin		–	–	–
11	Wang et al. (34)	Topical	Argireline (Acetyl hexapeptide-3; SNAP-25–derived synthetic hexapeptide)		Argireline (0% in oil–water emulsion)	Synthetic hexapeptide	Applied to peri-orbital wrinkles twice daily for 4 weeks
			Placebo Topical Vehicle		–	–	–
12	Bolke et al. (9)	Oral	Short chain oligopeptides composed of 5 to 8, 9 to 15, and 16 to 26 amino acids	Oral collagen peptides (2.5 g/day) with vitamin C (80 mg), zinc (3 mg), vitamin E (2.3 mg), and biotin (50 μg)	Oral collagen peptides (fish-derived, type I) with micronutrients	Fish collagen (type I)	2.5 g/day
				Oral collagen peptides (2.5 g/day) with additional fruit extract (acerola, 666 mg)			2.5 g/day
			Placebo: Isocaloric drink without collagen peptides		–	–	–
13	Czajka et al. (8)	Oral	Hydrolyzed fish collagen (type I)		Oral hydrolyzed fish collagen with vitamins (C, B-complex, D, biotin) and minerals (zinc, copper)	Fish collagen type I	10 g/day (50 mL drink)
			Placebo: Identical flavored drink without collagen peptides		–	–	–
14	Inoue et al. (3)	Oral	Collagen hydrolysates with Pro-Hyp and Hyp-Gly dipeptides		Oral collagen hydrolysates enriched with Pro-Hyp and Hyp-Gly	Fish gelatin	Low-dose (0.1 g/kg)
							high-dose (2 g/kg)
			Placebo: Maltodextrin		–	–	–
15	Ito et al. (35)	Oral	Collagen peptide and ornithine (CPO)		Oral collagen peptide with ornithine	Fish scales (Tilapia)	10 g collagen and 400 mg ornithine daily (30 mL drink)
			Placebo: Identical drink without CPO		–	–	–
16	Kim et al. (10)	Oral	Low-molecular-weight collagen peptide (LMWCP)		Oral low-molecular-weight collagen peptides	Sutchi catfish skin	1g/day
			Placebo		–	–	–
17	Miyeong et al. (36)	Oral	Collagen peptide (CPNS)		Oral fish-derived collagen peptides (low molecular weight)	Fish scales (Tilapia)	1.65 g/day (tablet formulation)
			Placebo		–	–	–
18	Taub et al. (37)	Topical	Alpha-defensin 5 and beta-defensin 3		Topical formulation with synthetic biomimetic peptides (α-defensin-5 and β-defensin-3)	Synthetic (α-defensin-5 and β-defensin-3)	–
			Placebo		–	–	–
19	Kim et al. (38)	Oral	Low molecular weight collagen		Oral low-molecular-weight fish collagen peptides	Fish scale collagen	2 g/day (500 mg tablets)
			Placebo: cellulose (40.0%) and maltodextrin (33.3%)				
			Placebo		–	–	–

BCP, bioactive collagen peptides; CH, collagen hydrolysate; CPO, collagen peptide and ornithine; CPNS, collagen peptide NS; CTP, collagen tripeptide; H-CP, high-dose collagen peptide; L-CP, low-dose collagen peptide; LMWCP, low-molecular-weight collagen peptide; NA/NR, not available/not reported; ONS, oral nutrition supplement; PRIMOS, phase-shift rapid in vivo measurement of skin; Ra, arithmetic mean roughness; Rmax, maximum roughness depth (maximum peak-to-valley height); Rz, mean roughness depth (mean peak-to-valley height); Rp, maximum peak height; Rv, maximum valley depth; Pro-Hyp, proline–hydroxyproline; Hyp-Gly, hydroxyproline–glycine.

The included studies reported outcomes such as hydration, elasticity, wrinkle reduction, and skin density; however, the methodologies used to obtain these measurements were not consistently described. In many cases, trials did not provide sufficient detail on whether validated, device-based assessments or subjective evaluations were employed. This lack of standardized reporting should be taken into account when interpreting the results.

### Results of individual studies

A total of 1,341 patients were included in 19 RCTs. Among the participants, the mean age was 50.2 years (±9.1). The study interventions included oral collagen peptides (*N* = 1,236; 92.17%%) and topical collagen formulations (*N* = 105; 7.8%). The results indicated significant improvements across various skin parameters, including skin hydration (*p* < 0.01), elasticity (*p* < 0.05), and wrinkle reduction, particularly in the crow's feet area (*p* < 0.01). Additionally, overall patient satisfaction was reported at 80%, with no severe adverse effects documented during the trials. The distribution of Fitzpatrick skin types among patients revealed Type II in 45 individuals (11.2%), Type III in 173 (43.0%), Type IV in 130 (32.3%), and Type V in 55 (13.5%). Fitzpatrick skin type was not reported for 20 patients (5.0%). The most frequently reported factors contributing to skin aging included sun exposure (*N* = 78; 19.4%) and lifestyle habits such as smoking (*N* = 43; 10.7%). Notably, a combination of sun exposure and smoking was reported by 10 patients (2.5%), while 8 patients (2.0%) cited genetic predisposition as a contributing factor.

### Efficacy of peptides in reducing signs of aging

**Figure 2:**
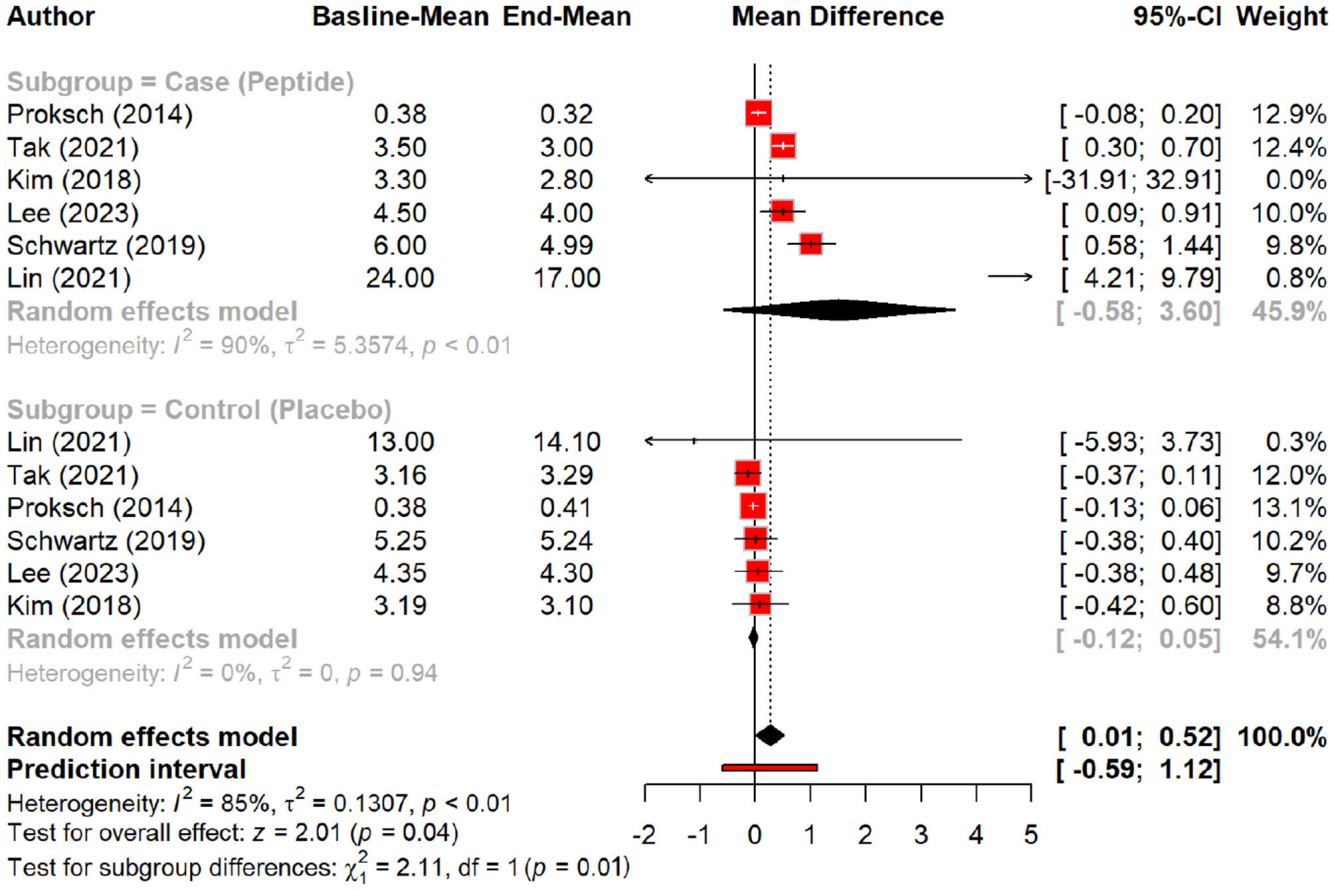
Effect of peptides on skin wrinkle reduction.

**Figure 3:**
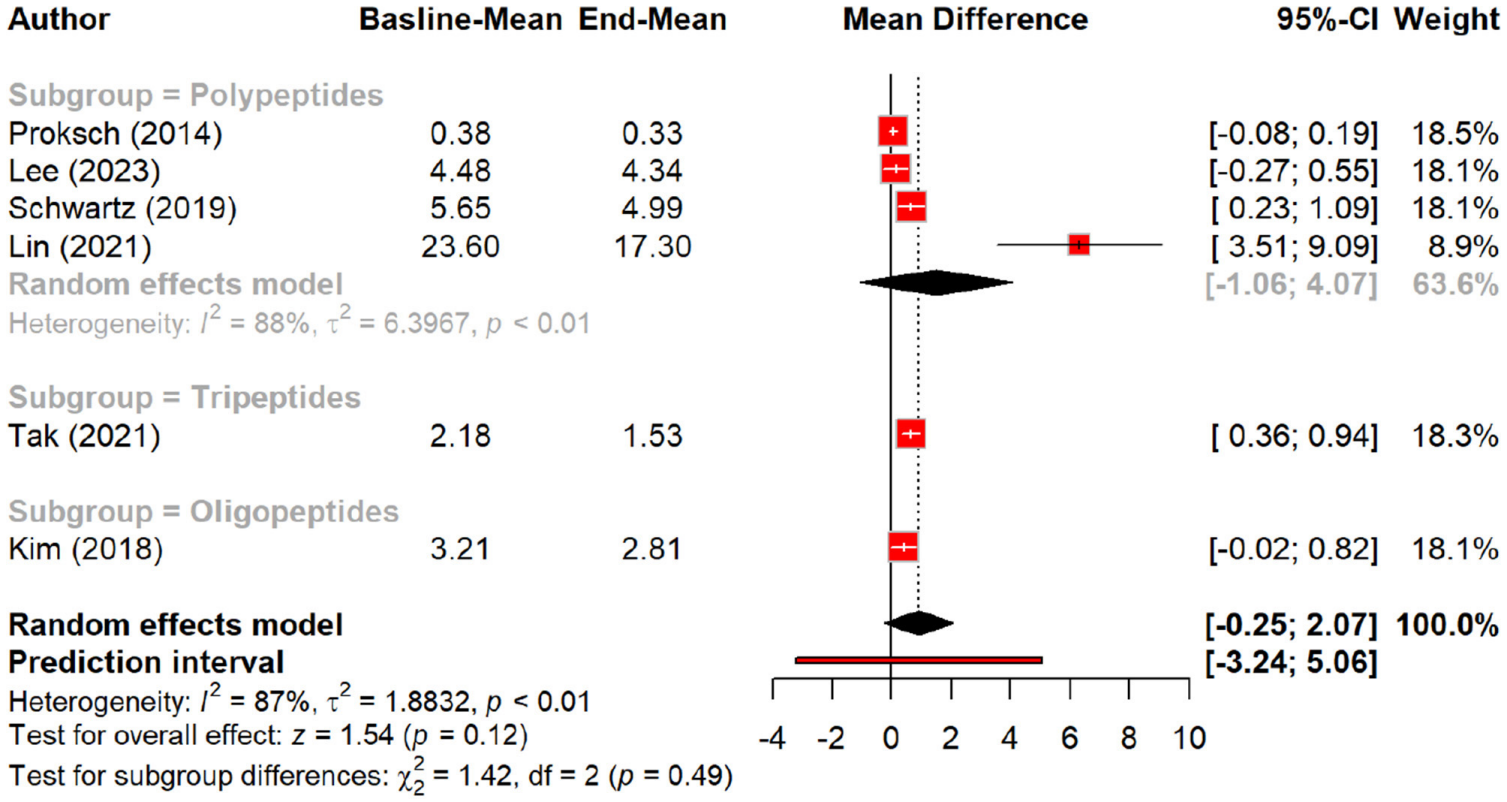
Subgroup analysis: skin wrinkle reduction degree by different types of peptides.

In this meta-analysis, we examined the efficacy of peptides in reducing signs of aging by evaluating their effects on various skin parameters compared to a placebo group. Peptides demonstrated a significant effect on wrinkle reduction, with a pooled mean difference (MD) of 0.27 (95% CI: 0.01–0.52, *p* = 0.04). The pooled effect suggests modest wrinkle reduction, but subgroup analysis demonstrates that oral polypeptides drive most of this effect (MD: 1.5, *p* = 0.01), whereas topical peptides showed a smaller, non-significant effect (Figures 2, 3).

**Figure 4:**
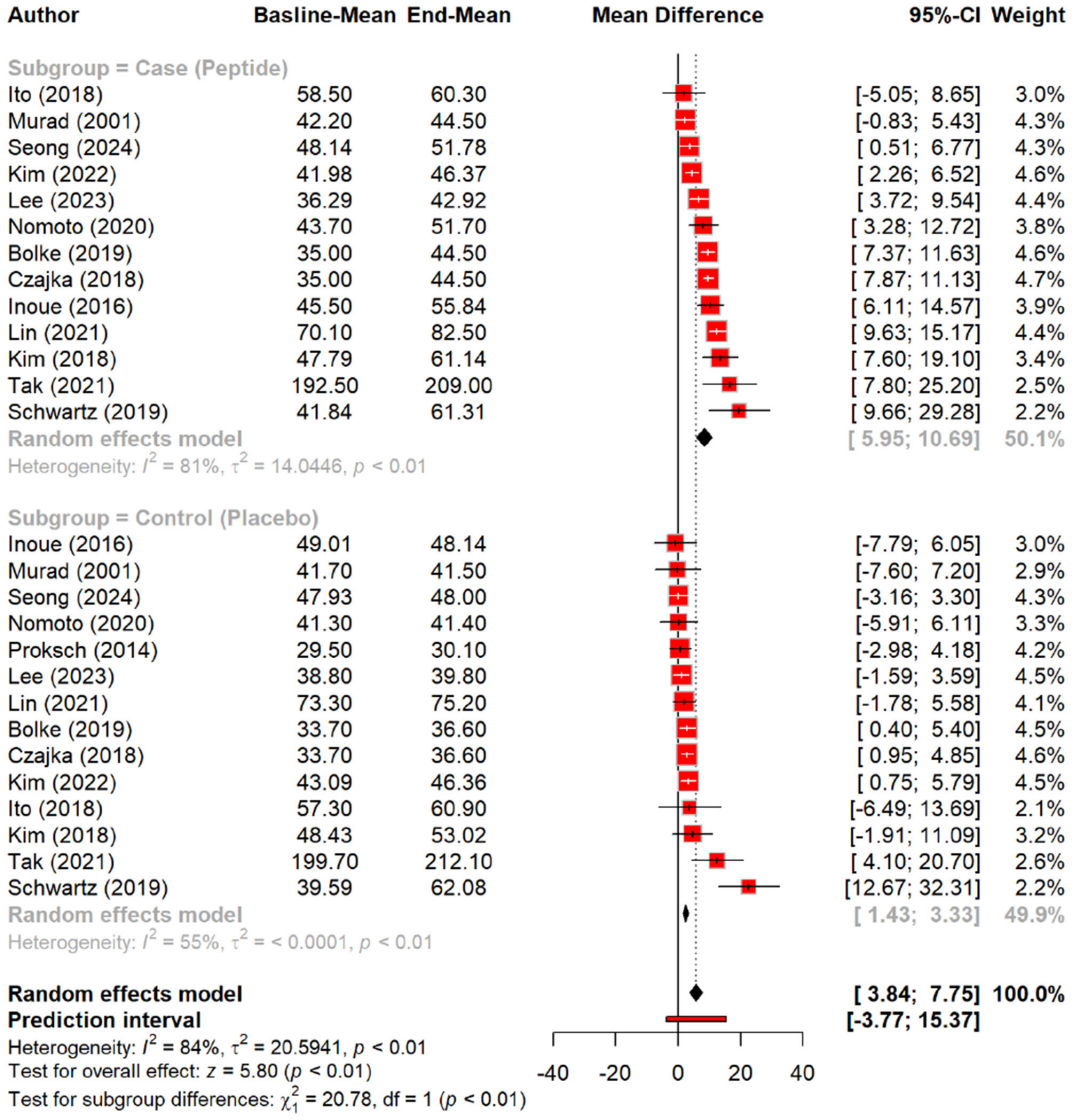
Effect of peptides on skin hydration status.

**Figure 5:**
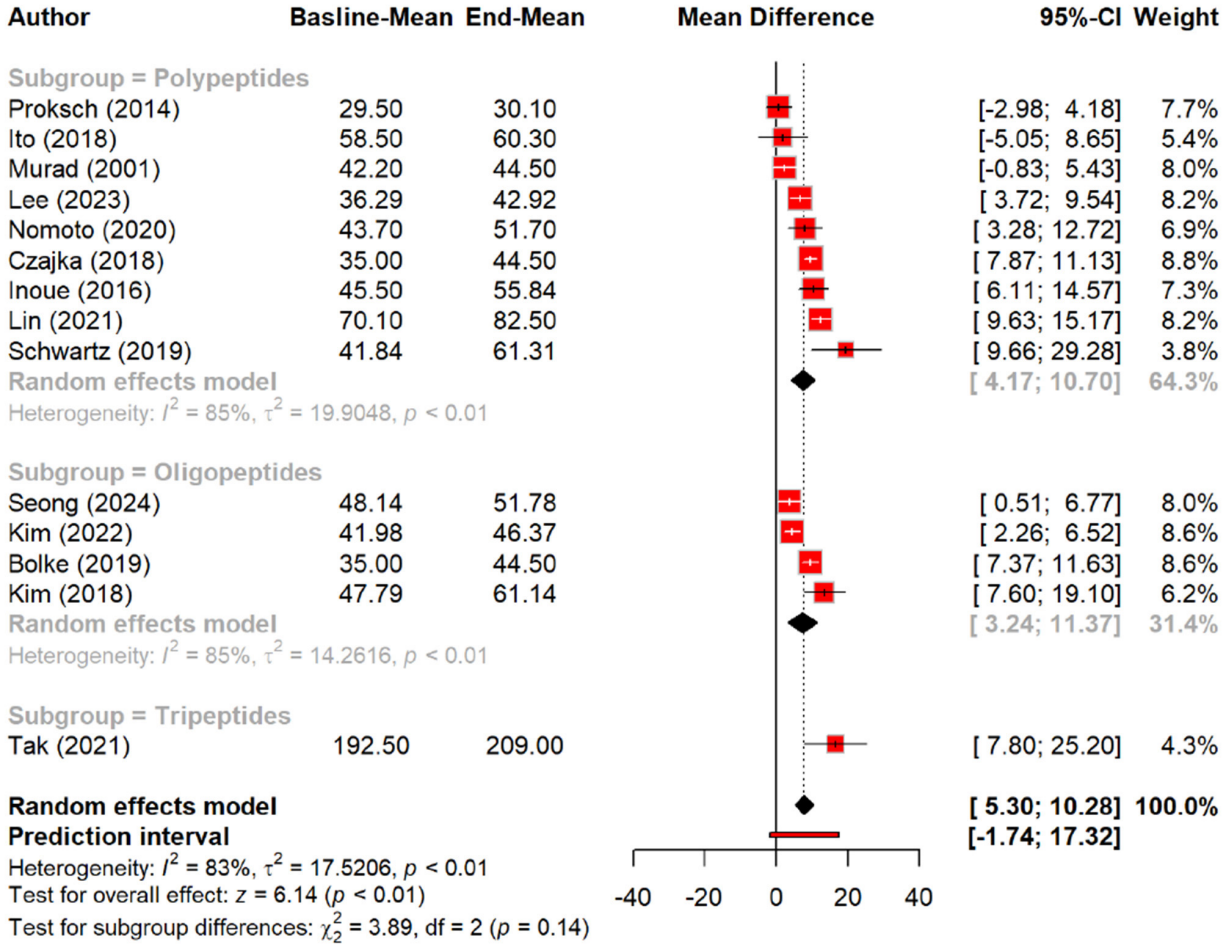
subgroup analysis: improvement in skin hydration (water content) by different types of peptides.

Regarding skin hydration, oral tripeptides showed the highest efficacy, resulting in a significant MD of 5.79 (*p* < 0.01), outperforming other peptides and placebo groups. Subgroup analysis reinforced this finding, with oral tripeptides achieving a greater increase in hydration (MD = 16.50) compared to other forms of peptides (MD = 8.31) and the placebo (MD = 2.37) (Figures 4, 5).

**Figure 6:**
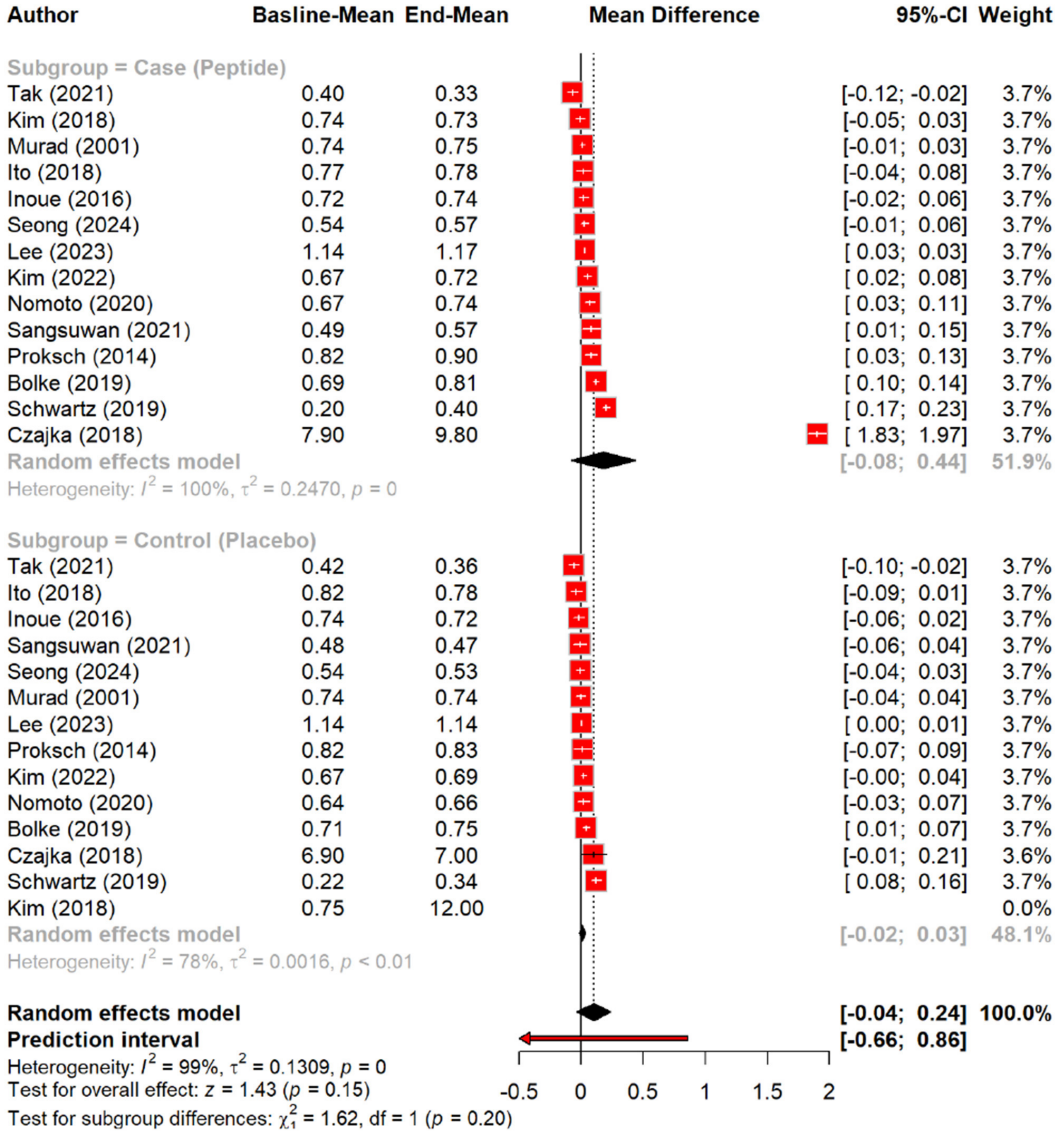
Effect of peptides on skin elasticity.

**Figure 7:**
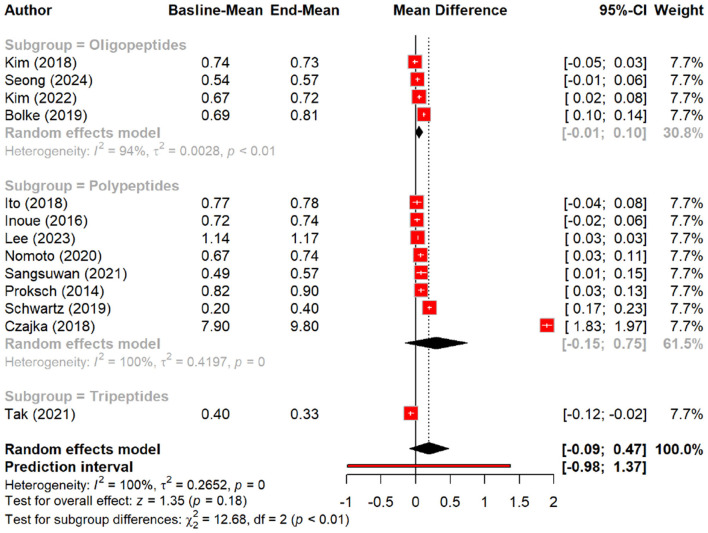
subgroup analysis: improvement in skin elasticity by different types of peptides.

The effect of peptides on skin elasticity was minimal, showing a non-significant MD of 0.09 (*p* = 0.15). However, subgroup analysis suggested a slight increase in elasticity with peptides (MD = 0.18) compared to the placebo (MD = 0.01, *p* = 0.20), with oral polypeptides exhibiting the highest effect (MD = 0.29) (Figures 6, 7).

**Figure 8:**
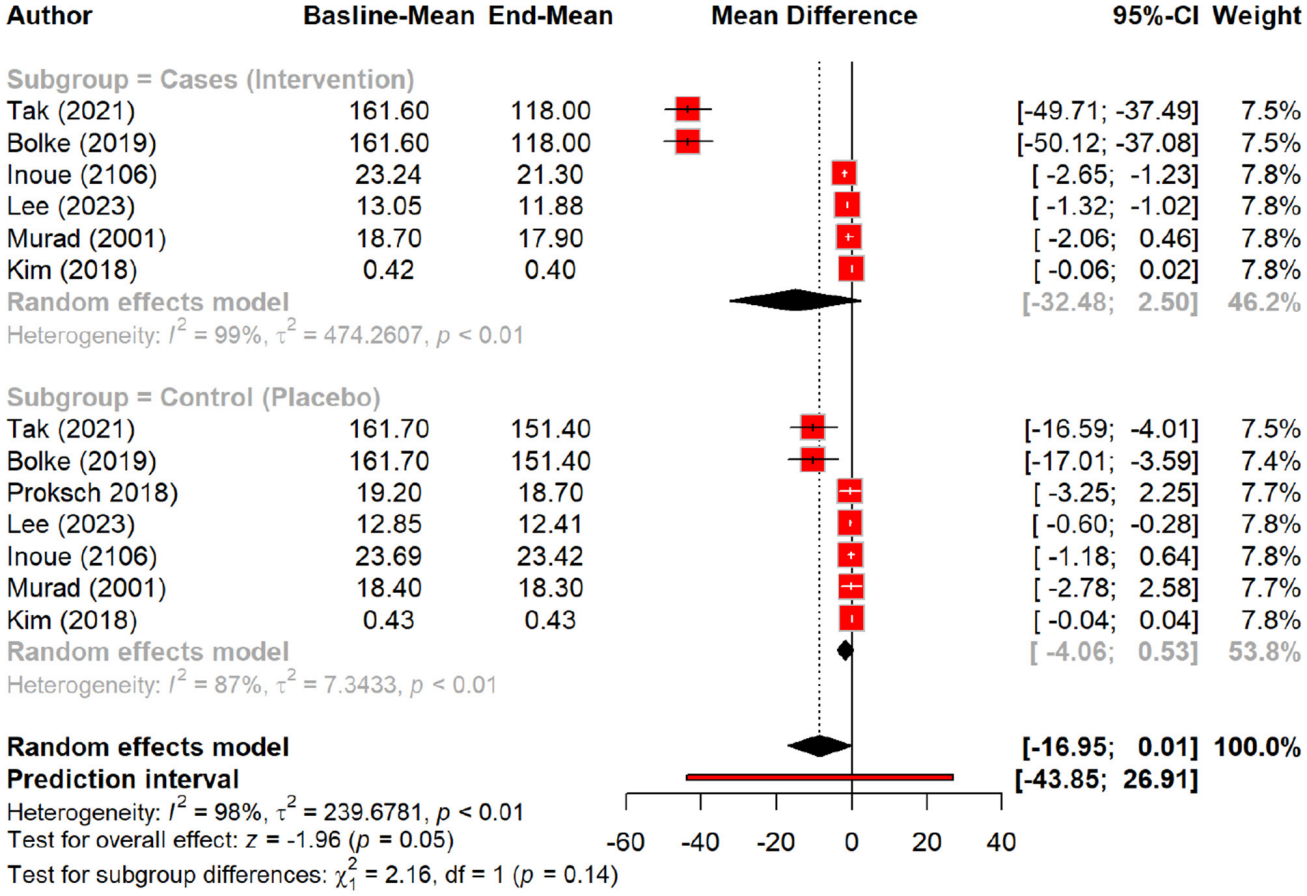
Effect of peptides on skin roughness.

**Figure 9:**
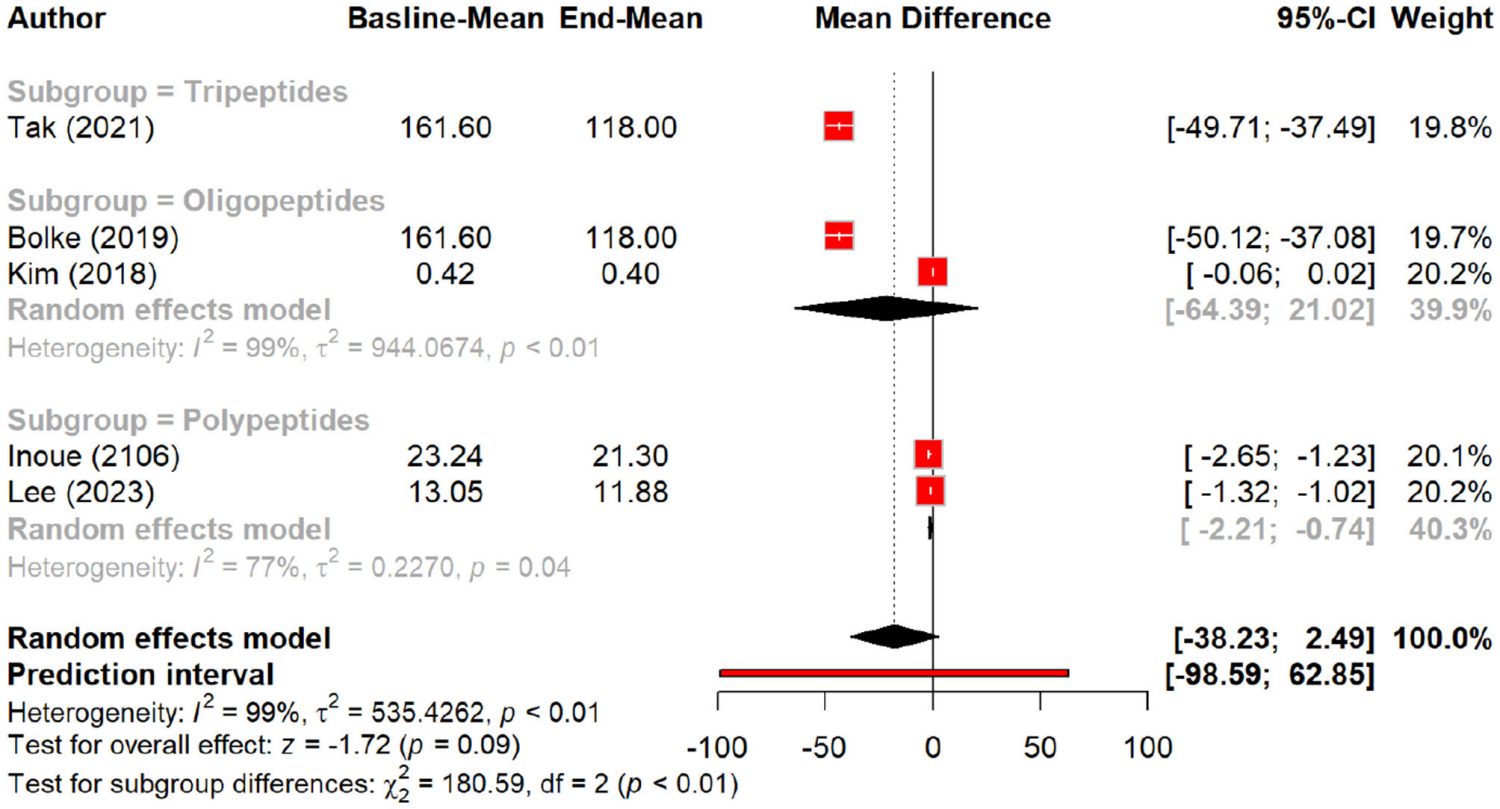
Subgroup analysis: reduction in skin roughness by different types of peptides.

**Figure 10:**
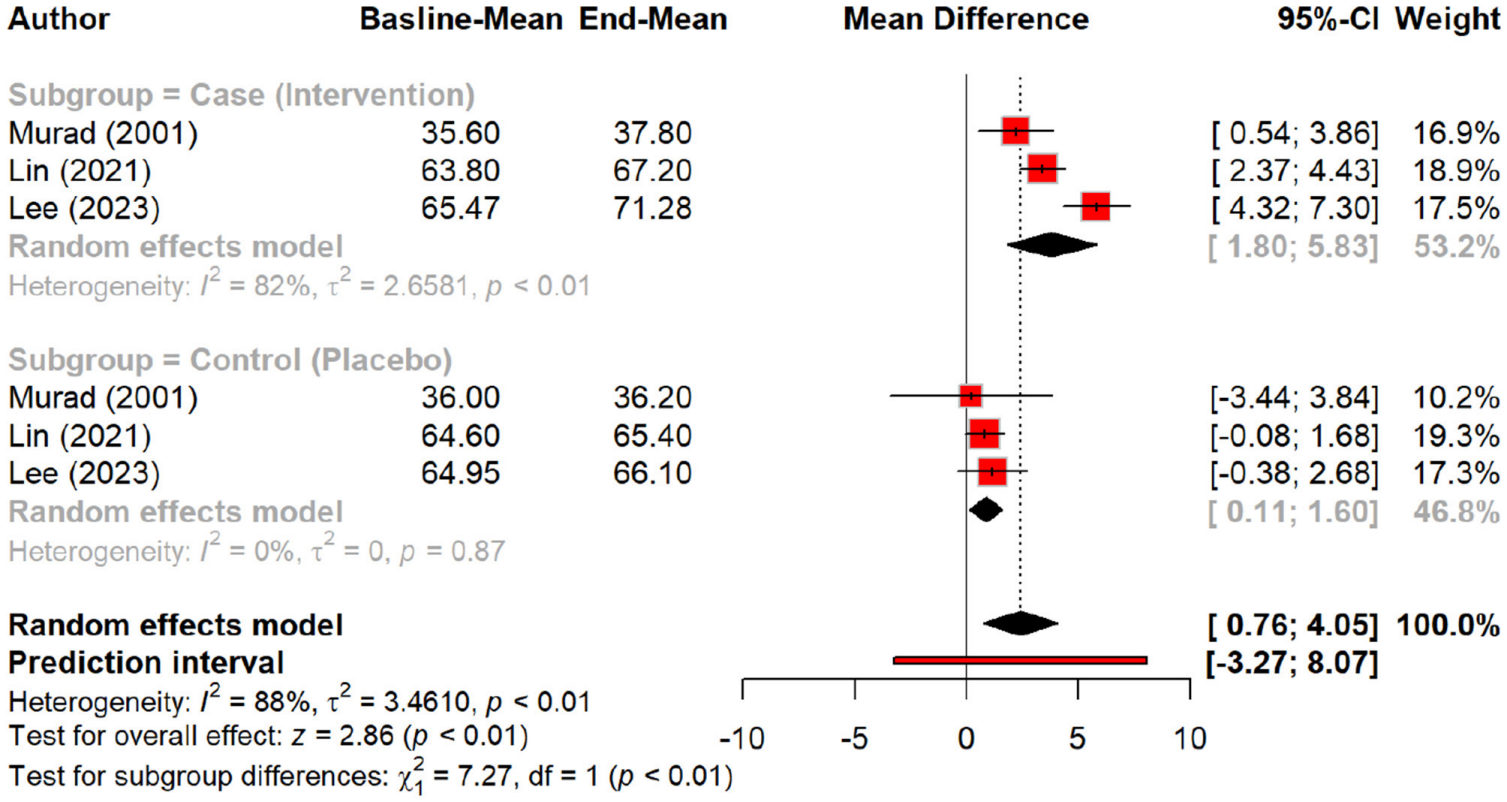
Effect of peptides on skin brightness.

**Figure 11:**
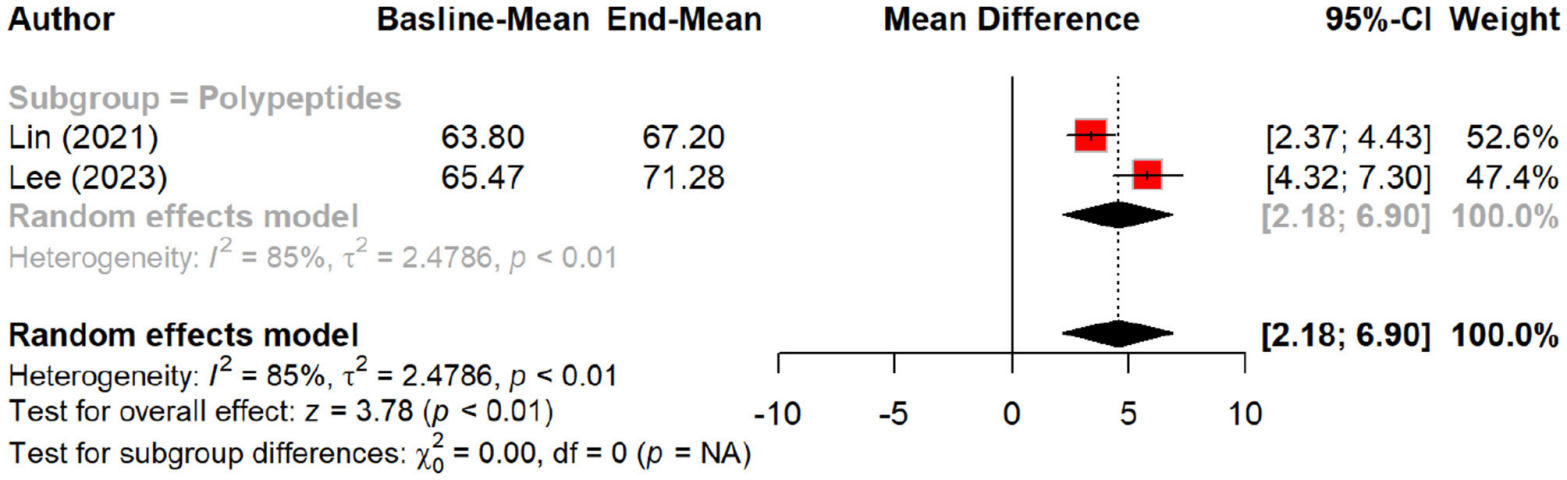
Subgroup analysis: improvement in skin brightness by different types of peptides.

**Figure 12:**
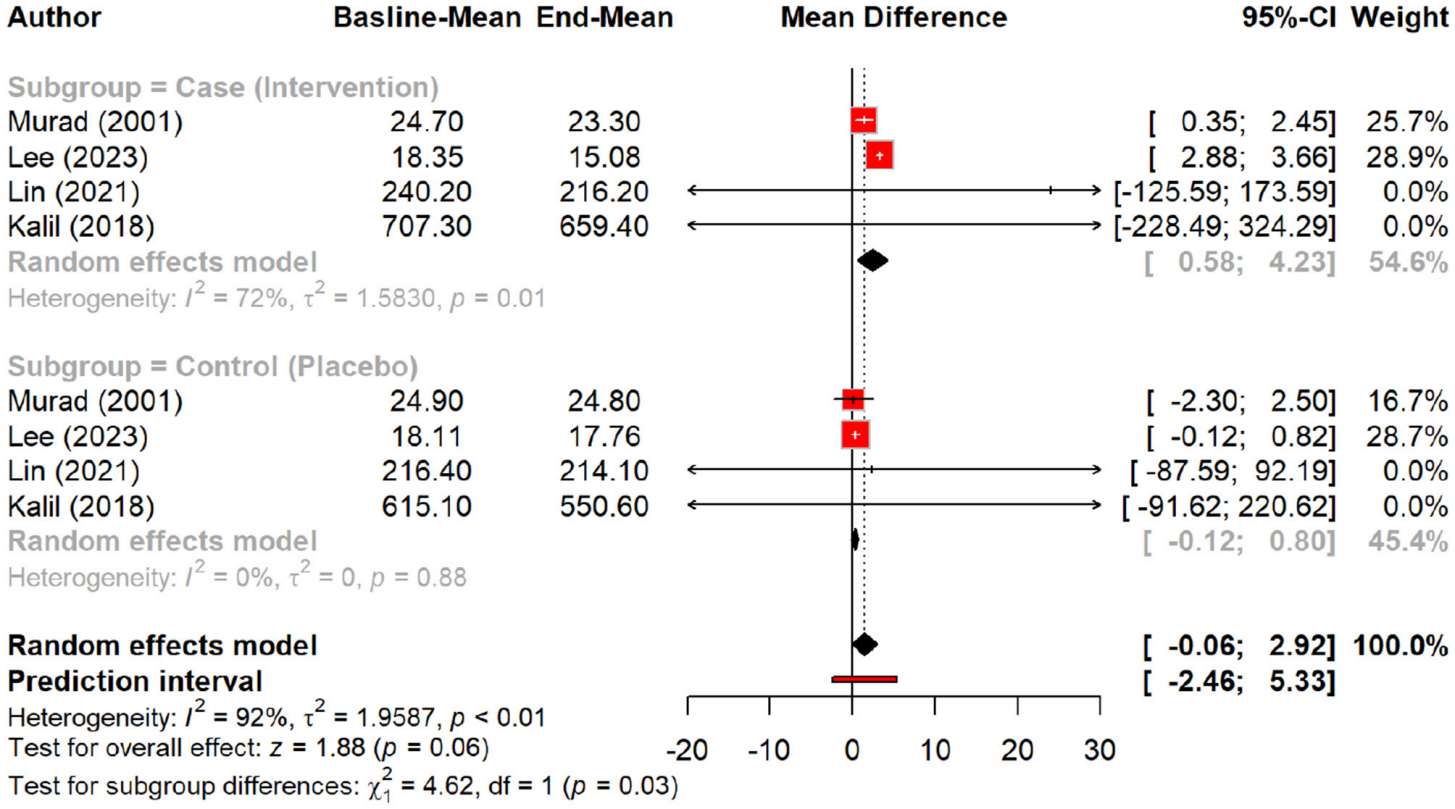
Effect of peptides on skin texture.

**Figure 13:**
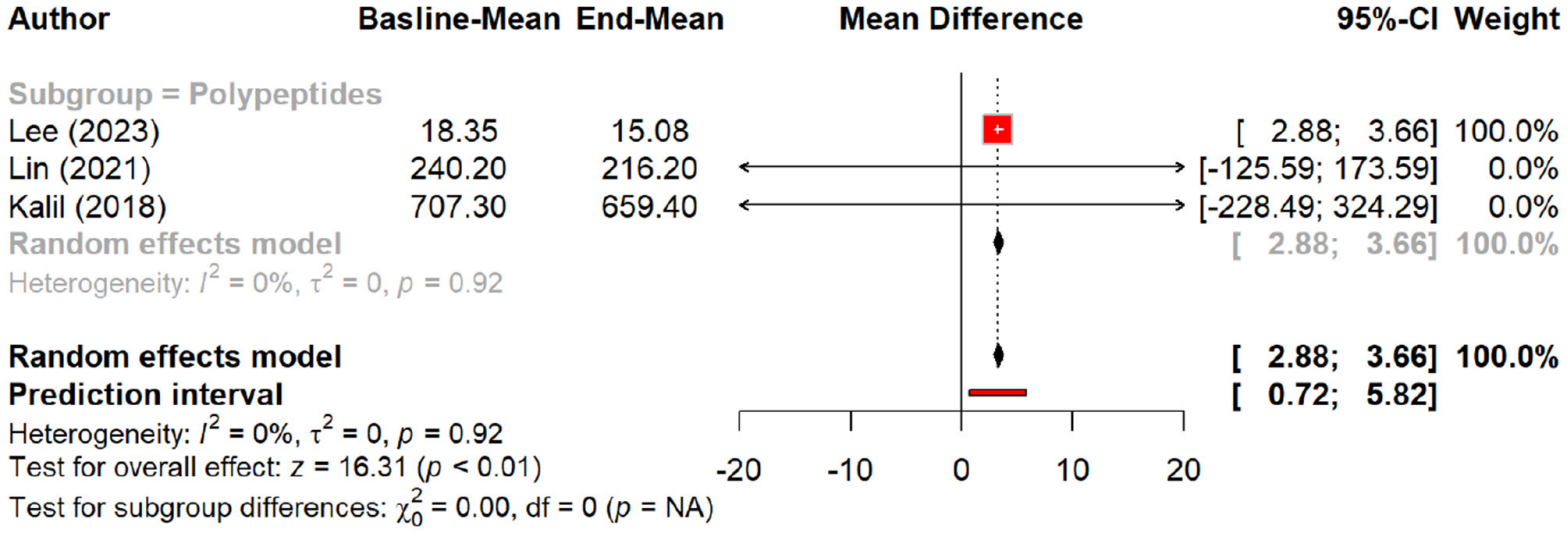
Subgroup analysis: improvement in skin texture by different types of peptides.

**Figure 14:**
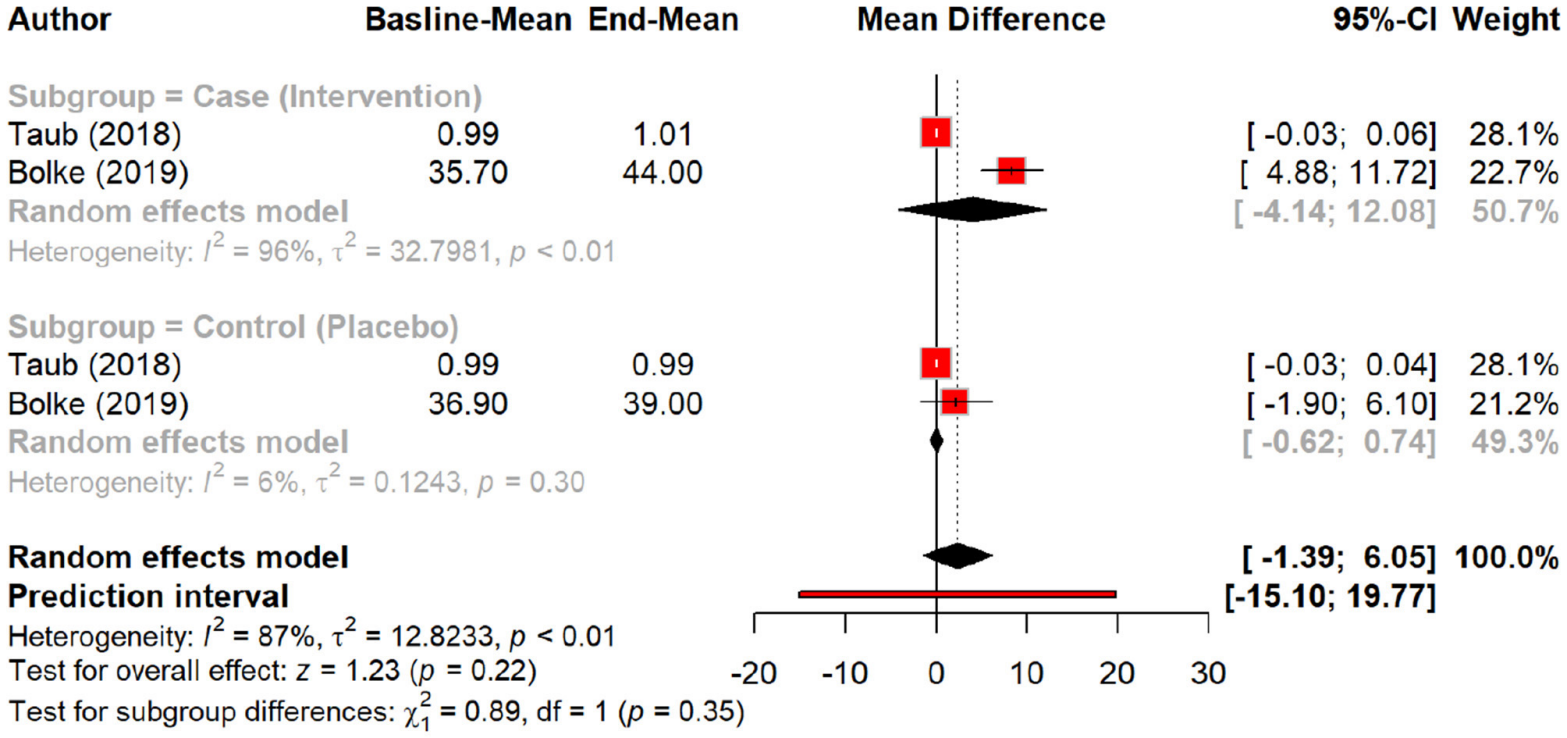
Effect of peptides on skin density.

**Figure 15:**
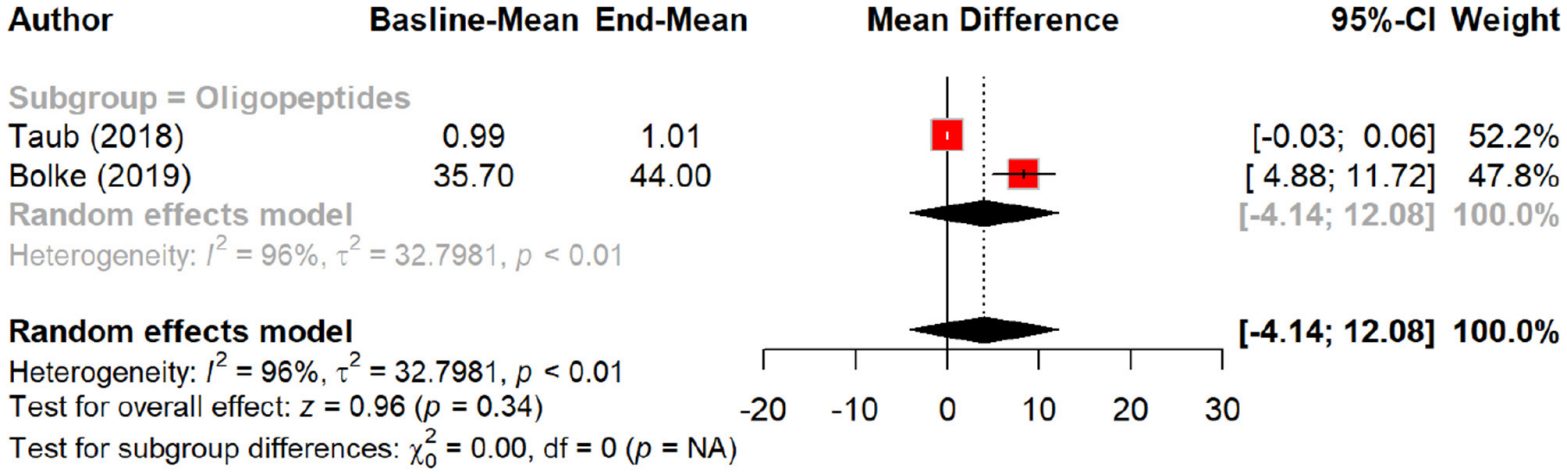
Subgroup analysis: skin density by different types of peptides.

**Figure 16:**
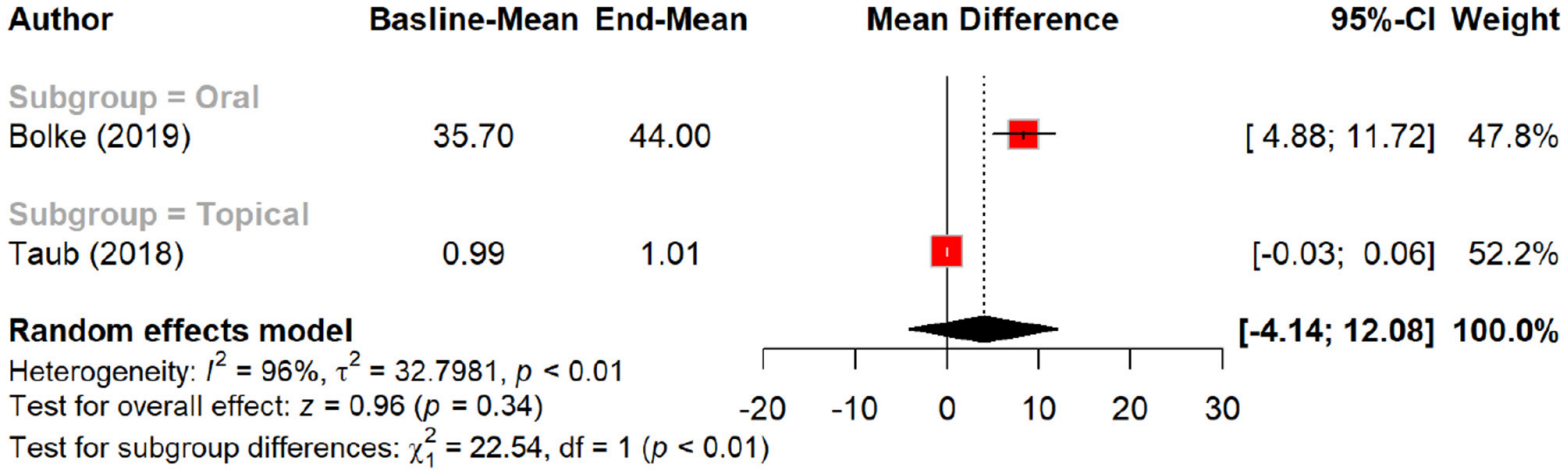
Skin density improvement by different routes of peptides.

Peptides significantly reduced skin roughness, with a nearly significant MD of −8.47 (*p* = 0.05). Subgroup analysis revealed a more substantial reduction in roughness with peptides (MD = −14.98) compared to placebo (MD = −1.76), particularly with oral tripeptides (MD = −43.60). Similarly, peptides notably improved skin brightness, with a significant MD of 2.40 (*p* < 0.01). Subgroup analysis highlighted a pronounced improvement with oral polypeptides (MD = 3.81) compared to placebo (MD = 0.85, *p* < 0.01) (Figures 8–16).

In terms of skin texture, peptides showed a non-significant improvement with an MD of 1.43 (*p* = 0.06). Subgroup analysis demonstrated a larger improvement in texture with oral polypeptides (MD = 2.40) compared to placebo (MD = 0.34, *p* = 0.03). Skin density exhibited a non-significant MD of 2.33 (*p* = 0.22), although subgroup analysis suggested a more notable increase in density with oral oligopeptides (MD = 3.97) compared to placebo (MD = 0.06, *p* = 0.34). Notably, peptides showed a significant effect on skin density when administered orally, but their topical application had minimal impact. Table 4 summarizes the results of meta-analysis of the secondary outcomes.

**Table 4:** Results of meta-analysis on secondary outcomes summary table.

**Outcome reported**	**Effect size**	**95% confidence interval**	***P*-value**	***I* ^2^**
Skin hydration	5.79	3.84–7.75	< 0.01	84%
Skin elasticity	0.09	0.04–0.24	0.15	99%
Skin roughness	−8.47	−16.95–0.01	0.05	98%
Skin brightness	2.40	0.76–4.05	< 0.01	88%
Skin texture	1.43	−0.06–2.92	0.06	92%
Skin density	2.33	−1.39–6.05	0.22	97%

### Sensitivity analysis

**Figure 17:**
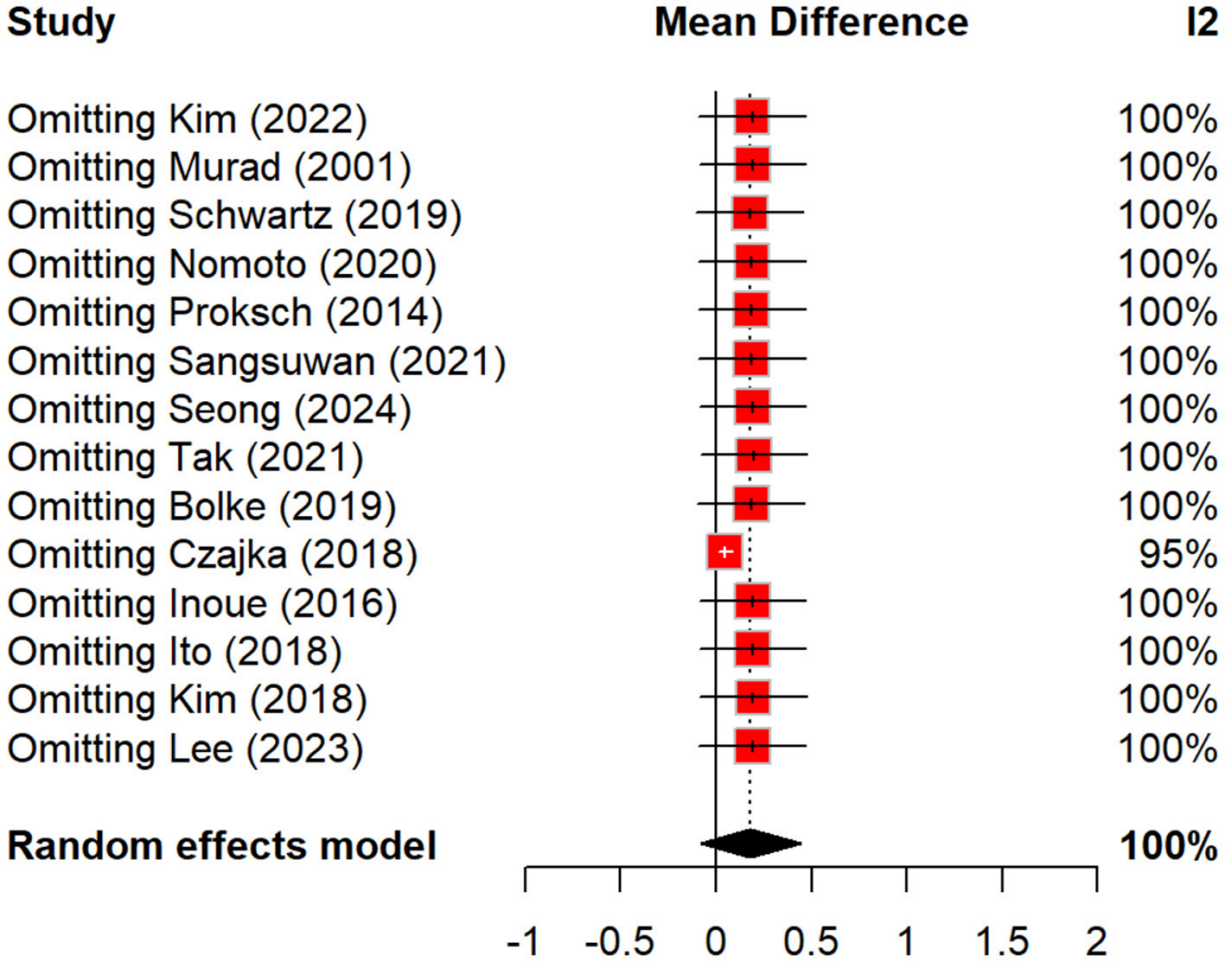
Sensitivity analysis assessing the influence of individual studies on wrinkle reduction outcomes.

A leave-one-out sensitivity analysis was performed to explore potential sources of heterogeneity (Figure 17). Exclusion of individual studies did not significantly alter the pooled estimates. The *I*^2^ statistic remained high at 100% for most exclusions, except for Czajka et al. (8), which reduced *I*^2^ to 95%, suggesting only a minor contribution to overall heterogeneity. These findings indicate that the substantial heterogeneity is most likely attributable to differences in peptide composition, dosing regimens, route of administration, intervention duration, and outcome measurement techniques rather than the influence of any single trial.

### Safety outcomes

The included studies reported minimal adverse events. Most participants tolerated peptides well, whether administered orally or topically. Mild side effects, such as gastrointestinal discomfort, were reported in a few cases following oral peptide intake. However, no severe adverse events or safety concerns were observed across any of the trials, indicating a favorable safety profile for peptide treatments.

### Methodological quality and risk of bias

**Figure 18:**
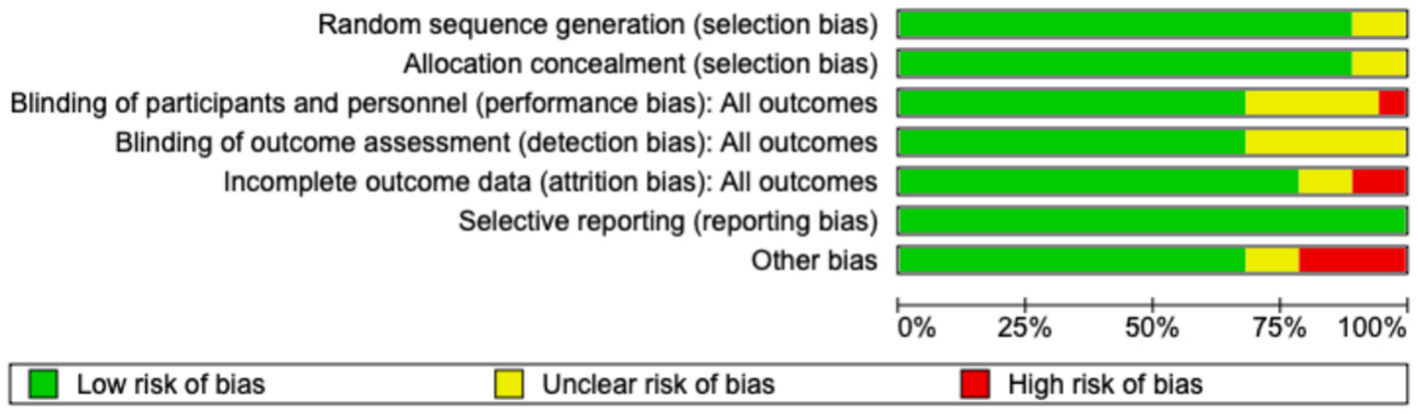
Risk of Bias assessment graphs.

**Figure 19:**
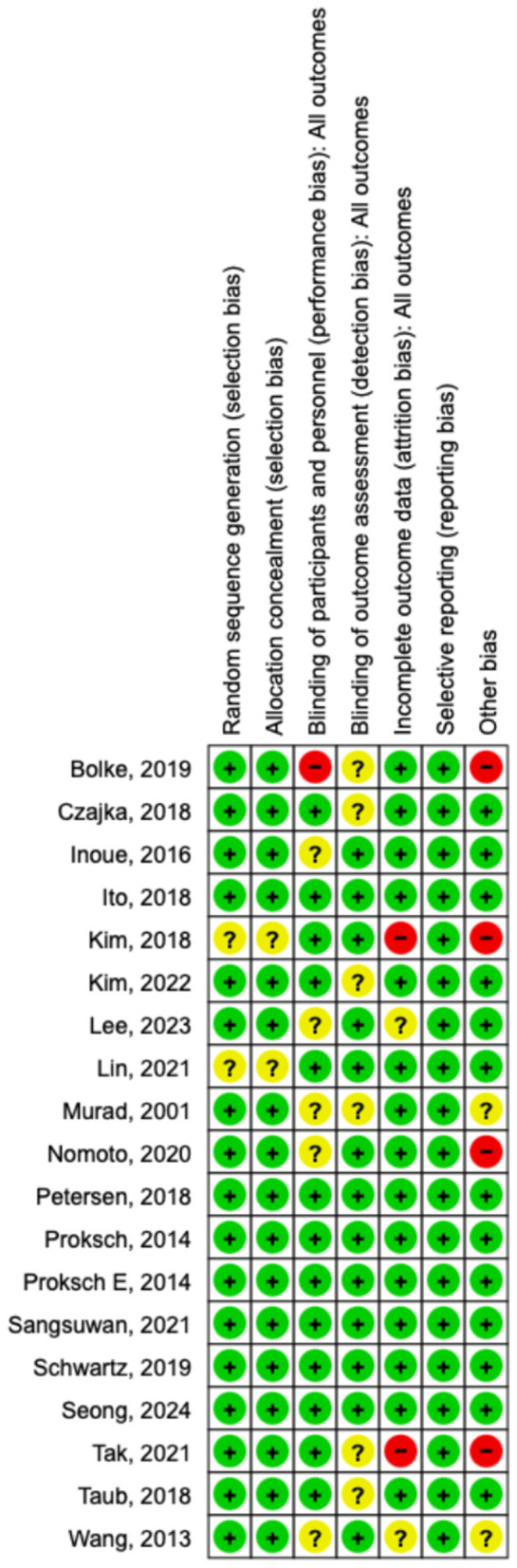
Risk of bias summary.

The risk-of-bias assessment indicated that most included studies, such as those by Bolke et al. (9), Czajka et al. (8), Inoue et al. (3), Ito et al. (35), Kim et al. (38), Lee et al. (36), Murad et al. (27), Nomoto et al. (29), Petersen et al. (30), Proksch et al. (24), Proksch E. et al. (31), Sangsuwan et al. (32), Schwartz et al. (28), Seong et al. (33), Tak et al. (11), Taub et al. (37), and Wang et al. (34), were at low risk in the domains of random sequence generation and allocation concealment. In contrast, Kim et al. (10) and Lin et al. (26) were rated as having unclear risk in these domains. The primary concerns related to blinding, with several studies demonstrating unclear risk, and Bolke et al. (9) identified as having a high risk of performance bias. Blinding of outcome assessment was also frequently unclear, indicating potential detection bias. Attrition bias varied, with high risk due to incomplete outcome data observed in Kim et al. (10) and Tak et al. (11). All studies demonstrated a low risk of selective reporting bias. The “other bias” domain demonstrated variability, with high risk identified in Bolke et al. (9), Kim et al. (10), Tak et al. (11), and Nomoto et al. (29). Figures 18, 19 provide a summary of the risk-of-bias assessment.

## Discussion

Peptide-based therapies have become increasingly prominent in dermatology for their targeted mechanisms of action and potential to reverse skin aging. Topical peptides are classified according to their delivery methods, such as creams, lotions, and serums, as well as by subtype, including signal peptides, carrier peptides, enzyme-inhibitory peptides, and neurotransmitter-inhibitory peptides. Each subtype is formulated to address specific mechanisms underlying skin aging (12, 13).

At the cellular level, signal peptides stimulate fibroblast proliferation and extracellular matrix (ECM) synthesis by activating TGF-β and MAPK signaling pathways, which increase collagen, elastin, and hyaluronic acid production (2). Other functional classes contribute to skin enhancement through complementary mechanisms: carrier peptides deliver trace minerals such as copper to support enzymatic collagen crosslinking; enzyme-inhibitory peptides protect collagen and elastin from enzymatic degradation; and neurotransmitter-inhibitory peptides reduce muscle contraction and dynamic wrinkle formation (14). Low-molecular-weight oral collagen peptides, which act as bioactive signal peptides, are absorbed through the intestinal barrier and distributed via circulation to the dermis, where they stimulate fibroblast activity and ECM regeneration (15). On the other hand, topical peptides reach target cells through skin barrier penetration, which may be enhanced by lipid conjugation or nanoparticle systems (4).

The reduction in skin wrinkles with peptide use showed a modest mean difference overall. The pooled effect suggests modest wrinkle reduction, but subgroup analysis demonstrates that oral polypeptides drive most of this effect. This highlights that the observed benefit is largely attributable to oral formulations, whereas topical formulations showed limited impact. Given the diversity in peptide formulations, concentrations, and molecular structures among the included trials, the findings should be interpreted with care. These differences likely contribute to the high degree of heterogeneity observed and limit the ability to attribute outcomes to any single peptide type. In addition, the treatment duration varied considerably across studies, ranging from short-term (4–8 weeks) to long-term (12–24 weeks) interventions, which may influence the degree of clinical improvement and further contribute to the variability in results. Subgroup analysis revealed a more pronounced reduction in wrinkles within the peptide group compared to placebo, though the comparison did not reach statistical significance. These findings are consistent with several other studies. For instance, Choi et al. (12) reported similar improvements in wrinkle reduction with peptide-based treatments, with both studies showing that peptides, especially topical formulations, could promote collagen synthesis and skin repair. This aligns with the findings of Han et al. (16), their research highlighted that peptides, delivered via nanocarriers, significantly increased collagen and hyaluronic acid synthesis in the skin, contributing to their anti-aging effects (13). However, our study is unique in demonstrating that oral polypeptides had a greater impact on wrinkle reduction compared to other peptide forms. This finding diverges from previous studies that predominantly focused on topical applications of peptides, such as Schagen et al. (18), which emphasized topical peptides as the primary driver of wrinkle improvement (16).

The effect of peptides on skin hydration was one of the most striking findings in this meta-analysis, with a significant mean difference favoring peptides. Subgroup analysis showed that the peptide group had a stronger positive effect on skin hydration compared to placebo. Notably, tripeptides, when taken orally, demonstrated the highest impact on hydration, surpassing other peptide types, suggesting that their smaller molecular size might facilitate better absorption and more efficient action in the skin. These findings corroborate earlier studies that have demonstrated the hydrating effects of peptides on the skin. For example, Miyanaga et al. (19) found that peptides, particularly in combination with moisturizers, enhance the skin's natural moisture retention mechanisms by increasing the levels of natural moisturizing factors (NMFs) in the stratum corneum (17). Moreover, Fisher et al. (20) found that synthetic peptides applied topically resulted in significant improvements in skin hydration, elasticity, and brightness highlighting peptides' potential for enhancing skin's luminosity and moisture levels (20).

Our results uniquely demonstrate that oral polypeptides may be more effective than topical formulations at enhancing skin brightness, potentially due to their systemic bioactivity, which could regulate melanin production at deeper levels. Unlike hydration, skin brightness showed significant improvement with peptide use, and subgroup analysis indicated a more pronounced effect in the polypeptide group. Previous research, such as Uchida (23), supports the role of peptides in improving skin tone and reducing pigmentation (23).

Regarding skin elasticity, our analysis found a non-significant mean difference, indicating a weak overall effect of peptides on skin firmness. This is consistent with previous evidence summarized by de Miranda et al. (21), who reported modest and heterogeneous improvements in skin elasticity, roughness, and wrinkle severity following oral collagen peptide supplementation. Our findings further suggest that these effects may be dependent on peptide type, as orally administered polypeptides showed the greatest, albeit non-significant, impact (MD = 0.29). Collectively, these results indicate that while peptide supplementation may offer limited effects on elasticity alone, it could provide more pronounced benefits for skin roughness and wrinkle severity.

The analysis of skin roughness revealed a nearly significant reduction in roughness with peptides, which is particularly relevant given that smoother skin is a key cosmetic goal for anti-aging treatments. Tripeptides, in particular, showed the greatest effect on roughness, supporting their potential use in smoothing the skin's surface. These findings align with research by Schagen et al. (18), which highlighted the role of peptides in enhancing cell turnover and skin barrier repair, leading to smoother skin over time (18). Moreover, Gibson et al. (22) also support this by showing that bioactive oral collagen peptides led to significant improvements in wrinkle volume and elasticity (22).

For skin texture, our results showed a non-significant improvement, but subgroup analysis suggested a larger effect in the oral polypeptide group compared to placebo, with a significant difference. This reinforces the idea that certain peptide types, particularly when taken orally, can have a notable impact on skin texture by promoting collagen synthesis and improving overall skin quality. A study by Hahn et al. (25) observed similar results with a palmitoyl peptide-based formulation, finding significant improvements in skin roughness and density after 4 weeks, thus aligning with the benefits of specific peptides for texture enhancement (25). Our findings should also be considered in light of heterogeneity across included trials. Variations in peptide type, dosage, intervention duration, and non-standardized outcome assessments likely influenced effect sizes and limited comparability between studies. Pooling validity is further compromised by methodological weaknesses in the included RCTs, particularly inadequate blinding and inconsistent outcome assessment methods. Our sensitivity analysis (Figure 17) further confirmed that no single study disproportionately influenced the pooled results. The *I*^2^ values remained consistently high, except when Czajka (8) was excluded, which only slightly reduced heterogeneity. This indicates that heterogeneity likely stems from broader methodological and clinical differences across studies rather than the effect of an individual outlier.

Another limitation is the imbalance in study routes, with 17 studies assessing oral peptides and only 2 evaluating topical formulations, which restricts the ability to directly compare their relative efficacy.

Finally, our analysis of skin density revealed a non-significant mean difference, with a slightly greater increase in the peptide group compared to placebo. Although this effect was not statistically significant, peptides taken orally showed a stronger impact on skin density than topical applications, likely due to better penetration and systemic effects. Oligopeptides, in particular, demonstrated some potential for increasing skin density, suggesting their use could be further explored in future studies. The greater impact of orally administered peptides on skin density may be attributed to their systemic bioavailability. Oral peptides, especially in low-molecular-weight forms, are absorbed in the gastrointestinal tract and distributed through the circulation, allowing activity in deeper dermal layers. In contrast, topical peptides are limited by stratum corneum penetration, unless advanced delivery systems are used, which may account for their comparatively weaker effects.

While peptides demonstrate modest yet statistically significant benefits for hydration and wrinkle reduction, the clinical significance remains limited by high heterogeneity and methodological inconsistencies. The overall magnitude of benefit should therefore be interpreted with caution.

## Conclusion

Our systematic review and meta-analysis suggest that oral and topical peptides may provide beneficial effects in improving several clinical signs of skin aging, with favorable safety and tolerability profiles. Notable improvements were observed in hydration, wrinkle reduction, roughness, and brightness, while the effects on elasticity and density were less consistent. Nonetheless, the current evidence base remains limited by heterogeneity in peptide types, dosing, and outcome measurement methods. In addition, only two high-quality topical peptide studies met the inclusion criteria and were analyzed alongside predominantly oral trials to provide a complete overview of available evidence. This limited representation of topical formulations should be considered when interpreting results, as findings primarily reflect oral peptide efficacy. Therefore, while peptides appear promising as safe and non-invasive adjuncts in anti-aging therapy, the available data are insufficient to support definitive conclusions regarding their efficacy, particularly for oral formulations. Future well-designed RCTs with standardized methodologies and detailed reporting are needed to confirm these findings and clarify the role of peptide class, dose, and administration route. Additionally, future trials should aim to balance study designs across oral and topical formulations to enable direct head-to-head comparisons.

## Data Availability

The original contributions presented in the study are included in the article/supplementary material, further inquiries can be directed to the corresponding author.
